# Hyperthyroidism Masking Depression and Panic Disorder Symptoms During Pregnancy: A Case Report

**DOI:** 10.7759/cureus.50582

**Published:** 2023-12-15

**Authors:** Joud K Alsaeed, Aldana M Zayed, Zainab H Buhassan, Sara H Alhadrami, Yahya Naguib, Mariwan Husni

**Affiliations:** 1 Psychiatry, Arabian Gulf University, Manama, BHR; 2 Psychiatry, King Hamad University Hospital, Muharraq, BHR; 3 Physiology, Arabian Gulf University, Manama, BHR

**Keywords:** maternal well-being, unborn child well-being, hyperthyroidism, pregnancy, antenatal depression

## Abstract

Depression occurring during pregnancy, often known as antenatal depression, is a significant mental illness with negative impacts on the mother's health, the health of the unborn baby, and the general welfare of the family. The condition is linked to various negative pregnancy outcomes, such as preterm birth, low birth weight, elevated risks of gestational diabetes, and preeclampsia. The signs and symptoms of depression in pregnancy do not differ from depression at any time. Another condition that may resemble symptoms of antenatal depression is hyperthyroidism, which is characterized by increased levels of thyroid hormones. Excessive levels of thyroid hormones can impact mood, energy levels, and overall well-being. It is crucial to differentiate between symptoms related to thyroid function and clinical depression. This case report could serve as a comprehensive approach addressing the management of antenatal depression with hyperthyroidism. This often involves a multidisciplinary approach, which includes collaboration between obstetricians, endocrinologists, and mental health professionals.

## Introduction

The significance of addressing antenatal depression as a serious health issue is pivotal for positive pregnancy outcomes. Instrument-based screening, such as questionnaires with cut-off scores, is needed to facilitate the treatments for antenatal depression [1]. This is crucial since pregnancy can potentially trigger or worsen depression in certain women due to hormonal changes and pregnancy-associated stress [2]. Additionally, hormonal shifts during pregnancy might affect the thyroid gland, leading to conditions like hyperthyroidism or exacerbating pre-existing thyroid issues [3]. Antenatal depression and hyperthyroidism may have overlapping symptoms such as disturbed sleep, weight loss, and anxiety [4]. The interaction between hormonal fluctuations and mental disorders can be quite intricate, especially during pregnancy. The current case illustrates a 35-year-old woman previously diagnosed with Graves disease based on low levels of thyroid stimulating hormone (TSH), high levels of free triiodothyronine (fT3) and free thyroxine (fT4) levels, as well as the presence of thyrotropin receptor antibody (TRAb). She was experiencing symptoms of depression and panic disorder across her multiple healthy previous pregnancies. This case report focuses on her clinical course, management strategies, and the patient’s experience in dealing with depression and panic attacks during pregnancy.

## Case presentation

The patient is a 35-year-old pregnant woman who was referred to the psychiatric clinic in September 2023 due to increased episodes of sadness and crying. At that point, she was a known case of Graves disease on propylthiouracil 100 mg twice daily. She was five months pregnant (gravida 5, para 3+1), with previous preterm spontaneous vaginal deliveries and one abortion. Upon assessment, the patient appeared well-dressed, exhibited good hygiene, and displayed a range of emotions from sadness to euthymia. Although the patient was not fully forthcoming with a detailed history, she reported experiencing low mood, crying spells, and negative thoughts (hopelessness and death wishes, but not suicidal thoughts) over the previous month. She also reported feelings of sporadic palpitations, shortness of breath, and pacing occurring for 30 minutes twice weekly. She emphasized that these episodes had become more frequent in the past few days. The patient also reported loss of appetite and sleep disturbances with struggling to initiate sleep for three months prior to referral to the psychiatric clinic. However, she denied any family history of psychiatric illness and had not seen a psychiatrist before. Nevertheless, the patient disclosed that she had similar symptoms during the previous pregnancies, which passed without medical attention to her psychiatric status. A diagnosis of antenatal depression with panic disorder was made by an experienced consultant psychiatrist based on DSM 5 criteria [5]. The treatment plan involved starting sertraline (selective serotonin reuptake inhibitor (SSRI)) in a daily dose of 50 mg. The patient was fully informed about all potential risks or side effects of SSRIs during pregnancy. After two weeks and despite starting sertraline, her symptoms persisted. Consequently, a decision was made to increase the dose of sertraline to 75 mg per day. Two months later, noticeable improvements in her depressive symptoms and panic attacks were observed. She was continued on sertraline 75 mg/day, and carbimazole 15 mg/day was resumed.

Her mental health needs were consistently overlooked throughout her previous medical and obstetrics encounters. In 2016, when she was 27 years old and during her second trimester of third pregnancy, she presented with a two-year history of unexplained weight loss (8 kg) accompanied by palpitations. At that time, laboratory results showed a low level of TSH, with high levels of fT4 and fT3. TRAb antibodies were detected in the patient's blood, and the diagnosis of Graves disease was confirmed. There were no other significant active medical problems detected at that time. A daily dose of carbimazole 5 mg was initiated. In 2019, despite having a normal range of thyroid laboratory results, the patient reported a persistent episode of palpitations. Consequently, propranolol (10 mg twice daily), when needed, was added to her treatment.

## Discussion

Antenatal depression is considered one of the most prevalent psychiatric disorders. Research indicates that within obstetric settings, the detection rate of depressive disorders is a mere 26% [6]. As a result, a significant portion of pregnant women persist in silently enduring depression throughout their pregnancy. Identification for depression during pregnancy should be considered, as untreated maternal depression poses a higher risk of developing postpartum depression and suicidality. Moreover, it may also impact childhood development, including altered neurocognitive development, as well as an increased risk of mental disorders and cardiovascular diseases in adulthood [7]. Regional studies within the Arabian Gulf have shown varying prevalence rates. For instance, Qatar presents a prevalence of 20.6%, similar to Oman and Kuwait. In contrast, Saudi Arabia reports a significantly higher prevalence of 57.5%. This suggests potential sociodemographic influences contributing to the regional variations in depression rates among pregnant women. The biopsychosocial model best describes the etiology of depression during pregnancy, attributing its causes to multifactorial elements like hormonal changes, past mental health history, and low social support [8].

Panic disorder (PD) is characterized by intense episodes of anxiety. The available limited data suggests a prevalence of approximately 3.7% for panic disorder during pregnancy [9]. It is also suggested that pregnant women with PD are at risk of placental abruption, fetal distress, decreased nutrition, preterm birth, anemia, and decreased fetal growth. The onset of panic disorder during pregnancy is linked to a heightened risk of relapse in subsequent pregnancies. Interestingly, some women with panic disorder experience improvement in symptoms during the early postpartum period [9].

Hyperthyroidism affects approximately 2% of women, with a pregnancy-related incidence ranging from 0.1% to 1% [10]. Clinical manifestations of abnormal thyroid hormone levels can manifest as a variety of neurological or psychiatric alterations, although the causal relationship can sometimes be unclear. Depression is the primary psychiatric manifestation of hypothyroidism. On the other hand, hyperthyroid patients are more prone to displaying psychotic symptoms. However, Grave's disease commonly coexists with disorders including depression, anxiety, and mental disorders. [11].

The treatment for hyperthyroidism typically involves antithyroid medications, which are thought to be free from unfavorable effects on the nervous system, both neurologically and psychiatrically [12]. Conversely, bringing thyroid dysfunction under control and treating it to achieve a balanced euthyroid state might lead to an improvement in mental disorders. While endocrinological treatment may improve mental health symptoms, a notable proportion of patients still encounter altered mental states even after successful hyperthyroidism treatment [11]. This may indicate potential involvement of mechanisms other than hyperthyroidism per se, including the autoimmune component of Graves' disease. Addressing persistent psychiatric disorders post-normalization of thyroid function might require specific psychiatric treatment, potentially involving psychotropic drugs.

During pregnancy, hormonal changes, heightened stress levels, and other factors related to this period can potentially trigger or worsen mental illness in some women. Additionally, the fluctuating hormone levels during pregnancy can affect the functioning of the thyroid gland, leading to conditions like hyperthyroidism or exacerbating existing thyroid problems [13]. The correlation between thyroid function and psychiatric conditions has long been recognized. There are limited studies specifically focused on antenatal depression and hyperthyroidism. A previous study demonstrated an association between thyroid dysfunction and depression in pregnancy. In that study, depressed women in late pregnancy had significantly higher concentrations of free T4 and a strong tendency towards lower TSH concentrations compared to non-depressed women [14].

Recognizing that hyperthyroidism's symptoms can resemble mental illness is critical to avoid misinterpretation and misdiagnosis of antenatal depression. Distinguishing hyperthyroidism from depression or panic disorder remains challenging [7]. Early routine screening for mental health issues, including depression and other disorders, is vital to prevent symptoms persistence and serious implications for both the mother and the developing baby. Therefore, ensuring accurate diagnosis of mental health problems through early routine screening is essential to provide appropriate care and support for both maternal and child well-being.

## Conclusions

During pregnancy, mental health challenges like depression and panic disorder can coincide with hyperthyroidism, causing overlapping symptoms and potential misdiagnosis. The co-occurrence of psychiatric and thyroid diseases may be the result of common pathophysiological pathways, including disturbance in hormonal levels and autoimmunity. Routine and early screening for mental health issues during pregnancy could be a key to differentiating between thyroid-related symptoms and psychiatric conditions. This could help in the prevention of misinterpretation and enable comprehensive treatment. For this, multidisciplinary medical teams should be recruited.
